# Bacteriophages and Lysins as Possible Alternatives to Treat Antibiotic-Resistant Urinary Tract Infections

**DOI:** 10.3390/antibiotics9080466

**Published:** 2020-07-30

**Authors:** Trinidad de Miguel, José Luis R. Rama, Carmen Sieiro, Sandra Sánchez, Tomas G. Villa

**Affiliations:** 1 Department of Microbiology and Parasitology, Faculty of Pharmacy, University of Santiago de Compostela, 15782 Santiago de Compostela, Spain; joserodrama@gmail.com (J.L.R.R.); sandra.sanchez@usc.es (S.S.); tomas.gonzalez@usc.es (T.G.V.); 2 Department of Functional Biology and Health Sciences, Microbiology Area, Faculty of Biology, University of Vigo, 36310 Vigo, Pontevedra, Spain; mcsieiro@uvigo.es

**Keywords:** urinary tract infections (UTIs), uropathogens, phage therapy, lysins, phage-antibiotic synergy (PAS)

## Abstract

Urinary tract infections represent a major public health problem as the rapid emergence of antibiotic-resistant strains among uropathogens is causing the failure of many current treatments. The use of bacteriophages (phages) and their derivatives to combat infectious diseases is an old approach that has been forgotten by the West for a long time, mostly due to the discovery and great success of antibiotics. In the present so-called “post-antibiotic era”, many researchers are turning their attention to the re-discovered phage therapy, as an effective alternative to antibiotics. Phage therapy includes the use of natural or engineered phages, as well as their purified lytic enzymes to destroy pathogenic strains. Many in vitro and in vivo studies have been conducted, and these have proved the great potential for this therapy against uropathogenic bacteria. Nevertheless, to date, the lack of appropriate clinical trials has hindered its widespread clinic application.

## 1. Introduction

Urinary tract infections (UTIs) are a major concern among patients and medical professionals, as, after respiratory tract infections, they account for the most common infections reported in primary care hospitals, and their management has become more difficult in recent years due to the increasing problem of antibiotic resistance. UTIs can affect the lower urinary tract (cystitis) or the upper urinary tract (pyelonephritis), causing great morbidity in women of all ages as well as in infant boys and elderly men [1]. The reasons why women are more prone than men to UTIs include a shorter urethra and practices such as the use of spermicides and diaphragms, which alter the normal microbiota and promote the colonization of the periurethral area with fecal bacteria. Older age is also a risk factor for both sexes, due to changes in the microbiome and immune system and the appearance of bladder or uterine prolapse, diabetes, and other risk-concomitant conditions [2].

UTIs can be clinically classified into uncomplicated, when the individual presents a healthy condition previous to the infection and has no structural abnormalities, or is complicated, when the patient is immunocompromised or presents risk factors such as urinary retention, urinary obstruction, calculi, renal failure, or pregnancy [3].

**Figure 1:**
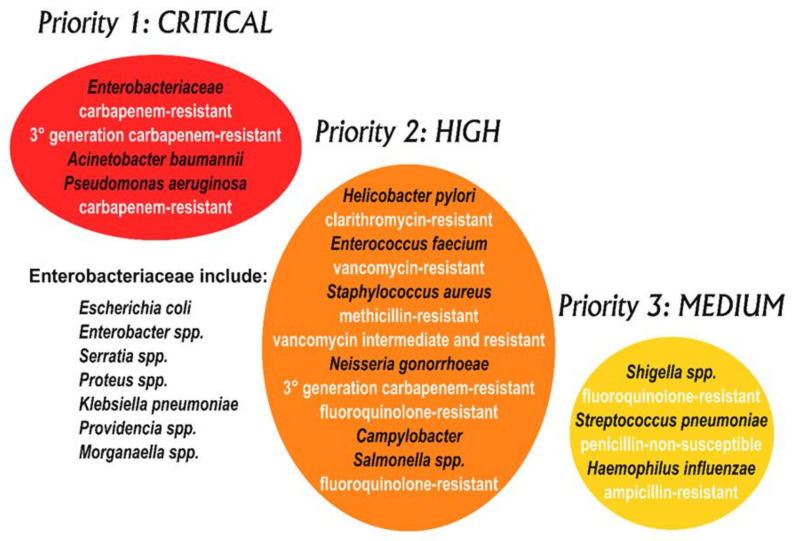
WHO priority pathogens list for R&D of new antibiotics [6]. Note that Mycobacteria are not included in this diagram, as they have, for some time, been considered separately as an absolute global priority.

Uropathogenic *Escherichia coli* (UPEC) is the pathogen most often found in both complicated and uncomplicated UTIs, followed by other bacteria belonging to both Gram-positive and Gram-negative groups—*Klebsiella pneumoniae*, *Staphylococcus saprophyticus, Enterococcus faecalis*, group B *Streptococcus* (GBS), *Proteus mirabilis*, *Pseudomonas aeruginosa*, *Staphylococcus aureus,* and the yeast *Candida* spp. [4]. The current treatments of choice for UTIs are based on antibiotherapy [5]. In recent years the management of UTIs has become difficult due to the emergence of several antibiotic-resistant strains of the most common bacteria infecting the urinary tract. Amongst the above-mentioned bacterial species, *S. aureus* has been listed by the World Health Organization as a high priority (priority 2) pathogen for research and the development of new treatments, whereas *P. aeruginosa*, *K. pneumoniae*, UPEC, and *P. mirabilis* are listed as critical or priority 1 pathogens (Figure 1) [6].

Uropathogens have specialized characteristics that enable them to colonize the urinary tract—the production of adhesins enables the pathogen to invade the bladder and renal epithelium, and the production of toxins, proteases, and siderophores allows the pathogen to obtain nutrients and iron from the host cells [4]. An important characteristic of the uropathogenic strains, that makes them more resistant to antibiotics and the host immune system, is their ability to form biofilms. These structures consist of communities of microorganisms embedded in self-produced extracellular polymers, which colonize both biological and abiotic surfaces such as catheters, posing a threat for many catheterized patients. Due to the increased cell density and the lack of oxygen and nutrients available to cells in the deepest layers of the biofilms, their metabolic activity is reduced, thus explaining the lower efficacy of antibiotics [7,8].

In recent years, the study of bacteriophages and their enzymes as an alternative to treat antibiotic-resistant UTIs has become of great interest. Although the optimization and usefulness of phage-based therapies is far from being a real fact, and no randomized clinical trial has shown positive results so far [9] they are being used as compassionate therapies due to some scientific evidence of their safety and beneficial activity [10].

The present article aims to review current advances in the use of phage-based therapies alone or in combination with existing antibiotics to handle UTIs and to discuss their possible usefulness and applications.

## 2. Current Treatment of UTIs

The current guidelines to treat UTIs, as revised by the Infectious Diseases Society of America (IDSA) and the European Society for Microbiology and Infectious Diseases (ESCMID), recommend the use of nitrofurantoin monohydrate/macrocrystals for the treatment of acute uncomplicated cystitis and pyelonephritis. Other antibiotics recommended depending on their availability and rates of local resistance, are trimethoprim-sulfamethoxazole (TMP-SMX), fosfomycin trometamol, pivemecillinam, fluoroquinolones (ofloxacin, ciprofloxacin, and levofloxacin), and β-lactam agents (amoxicillin-clavulanate, cefdinir, cefaclor, and cefpodoxime-proxetil) [11].

However, recent decades have shown the emergence of resistance against some of the antibiotics listed above [6]. In some regions, the uropathogenic strains resistant to TMP-SMX are more than 20% [12] and many UPECs are starting to produce extended spectrum β-lactamases, enzymes that cleave the characteristic ring of β-lactamic antibiotics, rendering them ineffective and worsening the already-threatening situation [13].

## 3. Brief History of Phage-Based Therapies

Bacteriophages, also called phages or bacterial viruses, were first envisaged in 1896, when the British bacteriologist Ernest Hankin reported that the waters of the Ganges and Jamuna Rivers had extraordinary antibacterial activity against *Vibrio cholerae*. Although Hankin could never discover its nature and origin, he described the occurrence of an antiseptic substance in these waters, which was thermolabile and could not be retained in porcelain filters [14]. When Twort and d´Hérelle, working separately, discovered the bacteriophages in 1915 and 1917, respectively [15,16], Hankin´s observation turned meaningful. Now, we know that those rivers contain a very high titer of phage particles that control, naturally, the spread of cholera in the regions they pass through.

Bacteriophages are by far the most-abundant biological agents on our planet and can be found in all environments, especially in the aquatic medium. They infect the host bacterial cell by binding to specific receptors located on the bacterial cell surface and releasing their genetic material (DNA or RNA) into it. Once the infection is ongoing, the phage can follow either the lytic or the lysogenic cycle. A lytic phage would use the host cells’ replication enzymes to make copies of itself and promote the bacterial lysis, which would release new infective viral particles. A lysogenic phage would integrate its genetic material into the bacterial genome resulting in a temperate phage. At the end, both cycles lead to the destruction of the host cell, so bacteriophages can be used against pathogenic bacteria [17].

The use of bacteriophages to combat bacterial infections dates back to the early twentieth century, and research and clinical practice on the topic has been performed continuously up until today in some countries of the former Soviet Union like Georgia and Russia [18]. Nevertheless, this therapy was not popular in other parts of the world, mainly due to the discovery and great success of antibiotics as chemotherapy agents [19]. Nowadays, bacteriophages are being used in the USA and EU as a compassionate therapy under the regulation of the article 37 of Helsinki Declaration [20], which limits its use to cases where there is no other possibility of intervention or all previous attempts were unsuccessful.

In the present scenario of the clinical practice, with pathogenic bacterial strains rapidly gaining resistance against many antibiotics, the Western world has turned its attention to this forgotten therapy, as an alternative to treat some infections, for which antibiotics are starting to fail.

There are two main approaches in the phage therapy—the one that uses the entire phage (either natural or genetically-engineered) to attack the pathogenic bacteria and the one that uses isolated phage lytic enzymes to promote e cell death.

## 4. Use of Natural Phages against Uropathogenic Strains

The approach based on the use of entire phages takes advantage of their self-replicating ability once they attack a bacterial cell. This leads to new infecting particles that will attack and destroy other cells in an exponential manner, ensuring their presence at the site of infection [21]. Some successful studies in vitro and in animal models show this therapy to be efficient against uropathogenic strains and other bacteria. In 2006 Capparelli et al. tested the efficacy of a phage isolated from cow manure in mice infected with *E. coli* O157:H7, and they were able to eliminate the infection from the mice within 48 h when the pathogen and the phage were injected simultaneously [22]. One year later, the same results were observed for 20 strains of *S. aureus*, including methicillin-resistant strains, using a similar procedure [23]. A study from Watanabe et al also using a single natural phage, showed significant improvement in survival of mice when *P. aeruginosa* was inoculated simultaneously with its natural phage KPP190 obtained from a highly-contaminated river in Japan. Nevertheless, no effect could be detected when the phage was administered some days after the infection was initiated [24].

One of the problems faced by phage therapy is the same as for antibiotics—the appearance of resistance. A promising strategy to avoid this phenomenon is to produce a cocktail of several phages, each one attaching to a different receptor on the cell, so that if one receptor is mutated there is another phage pointing to a different target. The more phages the cocktail contains the less likely resistances to all of them are to arise [25]. Several researchers have shown that cocktails delay the emergence of phage-resistant mutants. Tanji et al, working with *E. coli* O157:H7, tested in vitro the emergence of mutants when using two phages (namely SP21 and SP22) binding separately to different receptors. The appearance of resistant bacterial cells took place after 8 h and 6 h, respectively. Nevertheless, the combination of SP21 and SP22 in a cocktail delayed the emergence of resistant bacterial cells up to 30 h after administration [26]. Research in 2012 by Gu et al, analyzed the appearance of resistant strains of *K. pneumoniae* in infected mice after the administration of a three-phage cocktail and compared the results with the use of single monophages. The results showed that the phage cocktail reduced the bacterial mutation frequency and was more effective in rescuing the mice when compared with the monophage approach [27]. Similar results also in vivo were obtained by Chadha et al using five distinct phages for *K. pneumoniae* to treat burn-wound infections in mice. The cocktail containing the five phages was much more effective in reducing the bacterial load than any single phage [28].

Another limitation of using monophage therapy is the narrow host range. The use of cocktails broadens the spectrum of activity and would allow the targeting of different bacterial strains responsible for UTIs [25]. A study from Nishikawa et al obtained good results and a broader spectrum when they combined two T-even-related phages to treat multidrug-resistant UPEC in mice [29]. In a more recent publication Forti et al describe the successful treatment of *P. aeruginosa*-infected mice and wax moths (*Galleria mellonella*) using a cocktail of six different natural phages. The phages were first selected in vitro by assaying their activity against a collection of 40 *P. aeruginosa* strains. The selected combination was predicted to infect 97% of the total number of strains. The in vivo results in both animal models showed that the cocktail was much better than the monophage therapy and was very effective in destroying biofilms [30].

As stated before, uropathogenic strains tend to form biofilms in the urinary catheters. Those structures hamper the activity of the antibiotics and host defenses because of the spatial arrangement of the cells in the deepest layers. Due to the reduced oxygen intake that these cells receive, their metabolic activity is also very low, this disturbing the activity of antibiotics that target growing cells. So, biofilms can increase the minimum inhibitory concentration of antibiotics up to 1000-fold to overcome the infection. Additionally, the increased cell density in the biofilm promotes the appearance of resistance due to the enhanced possibility of resistance-gene exchange among the close cells [31]. So, biofilms do nothing but worsen the perspective of the existing therapies. Since about 75% of the UTIs are associated with the use of catheters [32], an effective system to attack the cells of the biofilm is needed. In this context, phage therapy can be useful to overcome the above-mentioned inconveniences. Some studies have revealed the effectiveness of single phages and cocktails when used against biofilm forming uropathogenic strains in vitro. Fu et al. obtained very good results with a single *P. aeruginosa* phage when it was applied before and at the same time as the infecting bacteria. However, they observed that the appearance of resistant strains took place after 24–48 h with the consequential re-growth of the biofilm. When a five-phage cocktail was used, the density of the biofilm formed at 48 h was reduced to 99,9%, thus indicating a remarkable delay in the emergence of resistance [33]. Two studies focus attention on *P. mirabilis*, an enterobacterium that causes catheter-associated UTIs by forming crystalline biofilms [32,34]. The results revealed that both individual phages and cocktails presented a strong anti-*P. mirabilis* biofilm activity and were highly stable. Although the phages have been proposed as good candidates to treat UTIs and catheter-associated UTIs, there is concern as to whether they would be able to keep their activity in vivo due to the flow caused by voiding or saline flush, or on the contrary whether they would be washed out of the system. Recent research by Blanco and Chen used a computational model that simulated the situation of a thin tube colonized by bacteria subjected to discontinuous flow. The results showed that as long as some bacteria are infected following inoculation with the phages, the therapy is effective even though all phages are completely washed out from the system [35].

## 5. Genetically-Engineered Phages

Despite the positive results shown so far, natural phages present some limitations for their use in clinical medicine that could be overcome by their genetic manipulation [36]: (i) Phages are typically specific for a limited set of related strains, but in practice, UTIs can be caused by a wide range of bacteria. As stated above, cocktails of several phages can be designed to broaden the spectrum. Nevertheless, regulatory approvals for such cocktails are difficult to obtain due to the great diversity of the containing phages [37]. (ii) A typical problem when using phage therapy is that, as with antibiotics, the fast lysis of the bacterial cells can lead to an adverse immune response in the host due to the release of toxic components of the cell wall, e.g., lipopolysaccharides (LPS) [38]. (iii) When the bacterial cells are included in a biofilm, they are surrounded by layers of extracellular polymeric substances (EPS) that prevent phages from reaching their targets [39]. (iv) It is not possible to prevent bacteria from developing resistance to the infection of natural phages [40].

So, the use of synthetic phages obtained by genetic engineering can help to overcome many of the above-stated limitations. Phages can be genetically manipulated by a wide variety of techniques—homologous recombination, bacteriophage recombination of electroporated DNA, in vivo recombination, clustered regularly interspaced short palindromic repeats (CRISPR)-Cas-mediated genome engineering, in vitro manipulation of phage genomes, whole-genome synthesis and assembly from synthetic oligonucleotides, yeast-based assembly of phage genomes, and cell-free transcription–translation systems [36]. There are some reports on phages engineered by these techniques, which have been successfully used against uropathogenic bacteria [41,42]. Lu and Collins produced an engineered phage expressing the *Actinobacillus actinomycetemcomitans* enzyme DSpB, which degrades the EPS present in biofilms formed by *E. coli* and *Staphylococcus*. The resulting phage promotes the lysis of the biofilm-forming bacteria simultaneously with the destruction of the EPS, this facilitating the phage particles to reach other bacterial cells [43]. A recent study by Møller-Olsen et al used the CRISPR-Cas technique to obtain a fluorescent phage specific for intracellular uropathogenic *E. coli* K1. They showed that both the *E. coli* strain displaying K1 capsule and the synthetic phage enter bladder epithelial cells via phagocytosis. The engineered phage was very efficient in killing the pathogen inside the human cells [44].

## 6. Phage Lytic Proteins

An interesting approach to control UTIs is the use of isolated phage lytic enzymes (PLEs) as antimicrobial molecules. These can be divided into two categories—endolysins and virion-associated lysins (VALs). Endolysins are lytic enzymes, expressed in the late replication cycle, that destroy the bacterial cell by attacking the peptidoglycan (PG) from within, allowing the virus progeny to spread. On the contrary, VALs are attached to the virion particle and degrade the cell surface from outside allowing the phage to inject its genetic material into the infected bacterial cell. Endolysins and VALs have been studied as good candidates to be used as antimicrobials because they are usually effective against a wide host range [45].

The early studies on endolysins and VALs have been directed to the attack of Gram-positive bacteria in vitro and in animal models, with good results in controlling antibiotic-resistant bacteria [46]. The cell wall of Gram-positive bacteria is easily degraded by PLEs due to the lack of a protective outer membrane (OM), whereas in Gram-negative bacteria the OM offers an effective barrier for the PG to be reached. As most of the uropathogenic strains belong to this group of bacteria, the application of exogenously-added PLEs in UTIs seem to be very limited [5]. Nevertheless, a small fraction of native phage lysins are effective against Gram-negative organisms. The first report on a highly-active therapeutic lysin against Gram-negative bacteria was lysin PlyF3017. It was selected from an array of naturally-produced *Acinetobacter baumanii* lysins and showed great activity against planktonic and biofilm *A. baumanii* cells, both in vitro and in vivo using a mouse bacteremia model [47]. As the authors suggested that the C-terminal regions of lysins could play a role in allowing access to the PG through destabilization of the OM, they obtained peptide P307, derived from the positively-charged C-terminal domain of PlyF307. P307 alone was sufficient to show a good in vitro activity against *A. baumanii* biofilms. A genetically-engineered derivative of this peptide showed excellent results in vitro and in vivo and presented a synergistic activity when combined with polymixin B [48]. In recent years, some studies have focused their efforts on combining PLEs with OM-disrupting agents and OM-permeabilizers to combat infections by Gram-negative UPEC, *K. pneumoniae*, *A. baumani,i* and *P. aeruginosa* [49,50,51,52].

## 7. Genetically-Engineered PLEs

**Figure 2:**
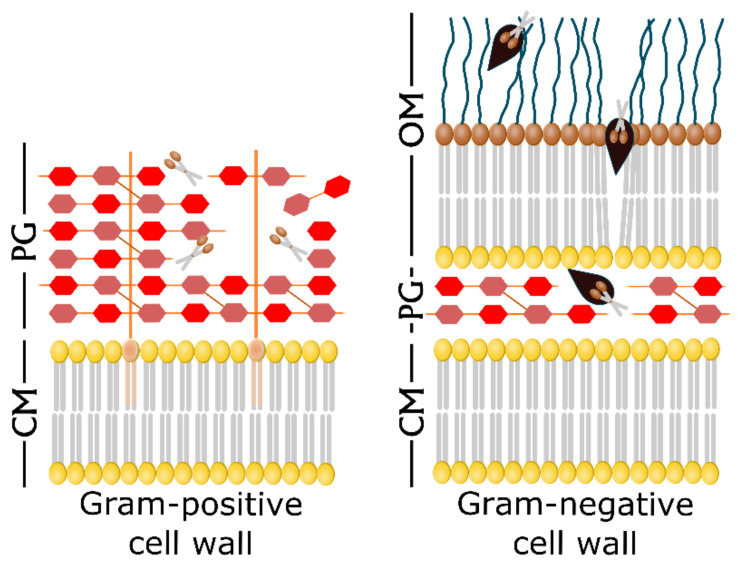
Exogenous addition of endolysins and Artilysin^®^s against the bacterial cell wall. The Gram-positive cell wall is directly accessible to exogenous endolysins (scissors) that cleave the peptidoglycan (PG) bonds, leading to the cell lysis (left panel). On the contrary, natural endolysins are not able to pass through the outer membrane (OM) of Gram-negative cells to attack the PG layers. Artilysin^®^s are engineered enzymes with an additional LPS-destabilizing peptide (black) that enables the endolysin to reach the PG and cleave it (right panel). Adapted from [54].

Engineering of PLEs has led to some improvements in the properties of these enzymes, facilitating them to penetrate the OM of Gram-negative bacteria (Figure 2). A successful strategy is the fusion of a PLE to a compound that binds a molecule on the OM, this allowing the enzyme to reach the PG. Lukacik et al. developed a synthetic enzyme by fusing the *E. coli* phage T4 lysozyme to a lytic toxin from *Yersinia pestis*. This molecule, called pesticin specifically binds to an OM transporter named FyuA. The resulting molecule from the coupling of phage T4 lysozyme to the FyuA-binding domain of pesticin is effective at killing cells expressing FyuA, and on the contrary it does not attack the natural gut microbiota [53]. The main limitation of this lysin is that it only targets cells expressing the FyuA receptor. On the other hand, one of the bacterial strategies to develop resistance is the mutation of receptors and other transport proteins. So, the mutation of the target receptor would render this strategy ineffective.

Another generation of engineered PLEs, namely Artilysin^®^s, has emerged to overcome the above mentioned problem. Artilysin^®^s are the resulting molecules from the fusion of a LPS-destabilizing peptide to an endolysin. This fusion can take place either at the N- or the C- terminus of the enzyme and does not affect its secondary or tertiary structure [54]. Several papers on this topic show how these engineered PLEs can be used against Gram-negative uropathogens such as *E. coli*, *K. pneumoniae,* or *P. aeruginosa*, including antibiotic-resistant strains. The first research report and mention of the term Artilysin^®^s is due to Briers et al [55]. The authors describe the modification of a modular endolysin by its fusion to a cationic nonapeptide and the further improvement of the construct to achieve better results. The so engineered enzyme presented excellent efficacy at destroying the PG of Gram-negative *P. aeruginosa* and *A. baumannii* both in vivo and in vitro, using a *Caenorhabditis elegans* model. The same group produced another highly efficient Artilysin^®^ named Art-175 able to kill all *P. aeruginosa* strains, including those that are multidrug-resistant and persisters [56]. Art-175 was later tested against colistin-resistant *E. coli* isolates proving its effectiveness and the lack of cross-resistance between colistin and Art-175 [57].

## 8. Phage Therapy in Combination with Antibiotics

For more than a decade it has been known that the phage infection of bacteria cultured with sub-lethal doses of antibiotic leads to an enhanced production of virulent phages. This phenomenon, first described and named phage-antibiotic synergy (PAS) by Comeau et al [58], can be observed as an enhanced size of the phage plaques. Although many authors reported PAS using different combinations of bacteria and antibiotics, Kim et al were the first to attribute it to a mechanism of “delayed lysis”. Based on their observations from a variety of bacterium–phage–antibiotic combinations, they concluded that stress-inducing substances like antibiotics may cause the elongation of the bacterial cells. The subsequent increase in the membrane surface area of the antibiotic-treated elongated cells reduces the holin concentration at the bacterial membrane [59].

Holins are phage-encoded proteins that aggregate to form pores in the cytoplasmic membrane of the host, which allows the endolysins to gain access to the PG [60]. In elongated cells the effective local concentration needed to form holin aggregates is not achieved at the right time to trigger lysis. This causes a delay in the lysis of bacterial cells and therefore an increase in the number of phages inside them.

Among the existing studies on PAS, there are no specific reports on UPEC, the most common uropathogenic bacterium. Nevertheless, we will point out some interesting pieces of research with other uropathogens, including biofilm-forming strains. A paper by Kaur et al reports the synergistic effect of treating methicillin-resistant *S. aureus* (MRSA) with its natural phage MR-5 and different types of antibiotics. Using the classical double-layer agar method, significant enhances in the plaque size were observed when using linezolid, tetracycline, or ketolides [61]. Ryan et al proved for the first time the effectiveness of PAS in biofilm control in vitro. Using the standard plaque assay, they demonstrated that the application of phage T4 and the antibiotic cefotaxime effectively destroys the cells of a biofilm-forming *E. coli* strain [62].

Recently, Kumaran et al designed a study to test if the sequence of application of antibiotic and phage was important to their efficacy. They used five different antibiotics and one phage against a biofilm-forming *S. aureus*. The phage and the antibiotics were applied in different ways—phage alone, antibiotic alone, both simultaneously, antibiotic followed by phage, and phage followed by antibiotic. Their results showed that infecting the biofilm cells with the phage prior to treatment with antibiotic causes the maximum reduction in the size of the biofilm [63].

The first report on PAS in *P. aeruginosa* is a paper by Knezevic et al. where the authors combine several antibiotics and phages. The best results were achieved after treatment with the antibiotic ceftriaxone and one of the assayed phages [64]. They detected a change in the cell morphology after treatment with ceftriaxone, which had been previously described for *E. coli* [58] and seems to be necessary for PAS. Some years later, a study by Chaudhry et al. analyzed the effect of the order of application on PAS using a biofilm-forming strain of *P. aeruginosa* [65]. As previously described for *S. aureus* [63] the authors found that the best results were achieved when the application of antibiotics followed the phage infection of the bacterial cells. In the most recent and exhaustive analysis of 25 different antibiotics and three phages against six strains of *P. aeruginosa*, Uchiyama et al. concluded that the best results of PAS for any of the strains used were achieved with phage KPP22 belonging to family Myoviridae genus *Pbunavirus* and the antibiotics piperacillin or ceftazidime [66]. There is a recent publication by Bao et al. reporting a case of synergism between a *K. pneumoniae* bacteriophage cocktail and a non-active antibiotic in vivo. The study describes the recovery of a patient with a recurrent UTI after the administration of five lytic phages and the antibiotics TMP/SMX. All *K. pneumoniae* strains isolated from the patient UTI were completely resistant to TMP/SMX [67].

## 9. Concluding Remarks

Increasing evidence on the emergence of multidrug-resistant strains of uropathogenic bacteria is making the scientific community look for clinical solutions, other than the search for new antibiotics, to this problem. Phage therapy is a promising alternative that has resulted in proved efficacy against UTIs both in vitro and in vivo using all the approaches discussed above—natural phages, phage cocktails, PLEs, engineered phages or PLEs, and phage therapy in combination with antibiotics. Nevertheless, an indispensable step for this therapy to be used in clinical practice is the evidence provided by validated clinical trials. Unfortunately, many clinical trials on phage therapy carried out so far have either been not adequately controlled or with a reduced sample size. Other studies have been interrupted due to lack of effectiveness [68]. To our knowledge, the only completed clinical trial regarding urinary infections is a phase II/III study in Georgia that targets “urinary tract infections in patients undergoing transurethral resection of the prostate” [9]. The design of this study using the phage cocktail named “Pyo” has been published, but not the final results [69]. A very recent phase I clinical trial in the USA is now recruiting to investigate the properties of phage cocktail LBP-EC01 in patients with lower urinary tract infection caused by *E. coli* [9].

Although there is enough evidence about the safety of phage therapy, it is important that the preparations meet legal requirements regarding sterility and absence of toxins [70]. Moreover, dose adjustment is vital to avoid problems derived from the toxicity of the components of the lysed cells’ walls, such as, in the worst scenario, toxic shock. Further data on this topic is needed to present robust results that support the phage therapy for clinical use. Although there are some reports on the recovery of patients treated with compassionate phage therapy, this has not yet been validated by clinical trials. So, larger error-free-designed clinical trials proving the safety and efficacy of phage therapy could propel its clinical use in the post-antibiotic era.
